# Hemolytic Uremic Syndrome Secondary to Scorpion Envenomation in a 7-Year-Old Boy from Southwestern Iran

**DOI:** 10.30699/ijp.2024.2024403.3272

**Published:** 2024-10-02

**Authors:** Nafiseh Mortazavi, Nakysa Hooman, Mitra Mehrazma, Yasaman Moradi, Parvin Aghavali

**Affiliations:** 1 *Aliasghar Clinical Research Development Center, Department of Pathology, School of Medicine, Iran University of Medical Sciences, Tehran, Iran*; 2 *Aliasghar Clinical Research Development Center, Department of Pediatrics, School of Medicine, Iran University of Medical Sciences, Tehran, Iran*; 3 *Hasheminejad Kidney Center, Department of pathology, School of Medicine, Iran University of Medical Sciences, Tehran, Iran*; 4 *Department of Pathology, School of Medicine, Iran University of Medical Sciences, Tehran, Iran*

**Keywords:** Hemolytic anemia, tubular injury, thrombotic microangiopathy

## Abstract

*Hemiscorpius lepturus* is a deadly scorpion species found in the tropical regions of the Middle East. Its venom consists of a complex mixture of peptides and enzymes, including the protease toxin hemiscorpius crolysin, the analgesic peptide, and the cytotoxic agent which attacks vascular low-body weight patients, and especially young patients, are prone to systemic complications such as nephrotoxicity, hemolysis, hepatotoxicity, and even death.

In this case report, we present a 7-year-old boy from city of Ahwaz in southwestern Iran, who was bitten by Gadeem (*H. lepturus*) and developed hemolytic uremic syndrome. After being stung, the patient developed hemolytic anemia, thrombocytopenia, and uremia in the subsequent days. The patient received supportive treatment, hemodialysis, and plasma exchange, and was discharged after 30 days of hospitalization.

## Introduction

Classification of scorpion stings is generally based on the severity of the symptoms, with Class I including local symptoms of redness, pain, and paresthesia, Class II including signs such as hypertension, urinary retention, and hematuria, and Class III referring to major cardiogenic, respiratory, renal and neurologic failure (1, 2). The analgesic peptide leptucin is present in the venom of the venomous species *Hemiscorpius lepturus*, so its stings are painless (3). The extent of its venomous effects largely depends on a patient's age and underlying health conditions. Younger patients and slim patients, because they have smaller body mass, are prone to systemic injuries such as disseminated intravascular coagulation, microangiopathic hemolytic anemia, and hemolytic uremic syndrome (1, 4). This report discusses a case of a child presenting hemolytic uremic syndrome caused by *H. lepturus* scorpion sting. The classic triad of thrombocytopenia, hemolysis, and uremia characterizes hemolytic uremic syndrome (4, 5), which is further categorized into typical (caused by *E. coli* infection O157: H7) and atypical HUS (6-8). Classic hemolytic uremic syndrome (HUS) involves laboratory findings of a diminished platelet count, anemia, a raised reticulocyte count, increased serum creatinine and urea, hematuria, and proteinuria (8). Also, a peripheral blood smear showed evidence of red blood cell fragmentation. ADAMTS-13 activity was normal, and coagulation test results were standard (8). In suspected cases of atypical HUS, it is necessary to determine the serum concentration of complement proteins (6, 7, 9). The present study reports on a seven-year-old boy, in which the diagnosis of hemolytic uremic syndrome was made by integrating clinical manifestations and laboratory findings. A renal biopsy explored histopathological and immunofluorescence to make a definitive diagnosis.

## Case Presentation

A 7-year-old boy from city of Ahwaz in southwestern Iran presented with bloody urine and loss of consciousness for a period of seven days. The cause was a scorpion sting of *H. lepturus* on his right arm. The incident occurred two days before appearance of the symptoms. On the first day of illness, the patient had bloody urine, respiratory distress, and weakness. At the emergency department of Golestan Hospital, Jundishapur University of Medical Science, Ahwaz, Iran, the patient received initial treatment including polyvalent scorpion antivenom, Cefazoline, and sodium bicarbonate in Dextrose water. Even after the beginning of daily hemodialysis therapy during his hospitalization, the relief afforded was insufficient, and the patient’s consciousness level, lower limb pitting edema, and coagulopathy all worsened. After seven days, the patient was referred to our department, Aliasghar Hospital, Iran University of Medical Science, Tehran, Iran, with ascites, an impaired ejection fraction, and a lowered level of consciousness (Glasgow Coma Scale Score of 11 out of 3-15). His vital signs upon admission were as follows: body temperature, 36.9°C; blood pressure, 137/101 mm Hg; pulse, 94 beats/min; respiratory rate, 20 breaths/min. His abdomen was not protected, and it was tender. Percussion disclosed changing dullness, which is a sign of ascites. Normal bowel sounds were observed. The right arm showed an area of ecchymosis and necrosis with local edema measuring 7×4 cm. At admission, hemoglobin and platelet counts were 9.4 g/dl and 99,000/mm³, respectively. Urine analysis revealed 3+ proteinuria and 3+ hemoglobinuria. The serum creatinine (Scr) and blood urea nitrogen (BUN) levels were 3.6 and 62.2 mg/dL, respectively.

**Fig 1:**
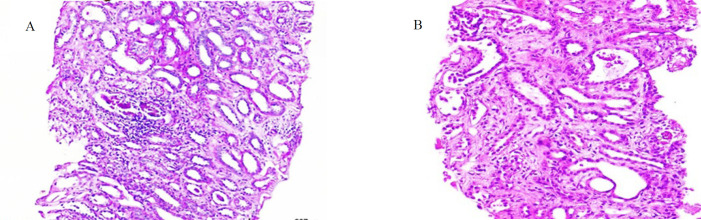
A & B: Interstitial edema and severe inflammatory cell infiltration (H & E; X400).

**Fig 2.**
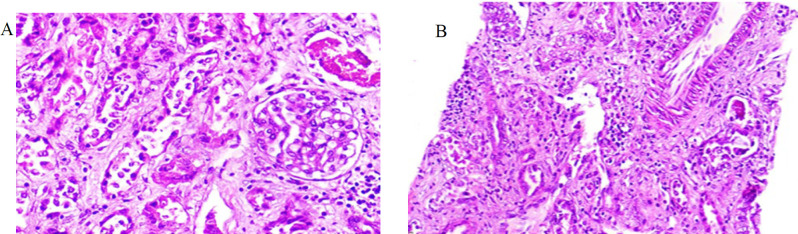
**A:**The glomerulus is rather than normal sized with mild mesangial hypercellularity. No spike, crescent, adhesion or fibrin in the bowman’s space (H&E; X 400).

LDH level 1989 IU/L, negative Coombs test, normal G6PD level. On a blood smear were fragmented erythrocytes. Prothrombin and partial thromboplastin time were within the normal range; blood gases revealed metabolic acidosis and respiratory alkalosis. The diagnosis from the patient's clinical presentation and blood test all pointed to hemolytic uremic syndrome (HUS). Immediate symptomatic treatment was initiated, along with alternate-day hemodialysis. Further, 600 ml of FFP was given daily over six treatment sessions, and the patient was treated with Prednisolone (5 mg per day), amlodipine (5 mg per day), and antibiotics. After 20 days of therapy, a renal biopsy confirmed the diagnosis. He took two cores containing up to 15 glomeruli of renal cortex and corticomedullary junction. The analysis showed the glomerular structure and renal tubule cells to be normal. They demonstrated an intact glomerular configuration and renal tubule cells. The interstitial tissue, marked by significant infiltration of inflammatory cells, includes lymphocytes, polymorphonuclear leukocytes (PMN), and a limited number of eosinophils (Figure 1) with observable areas of permeation between the tubular epithelial lining resulting in tubulitis (Figures 1B & 2A).

**B:** The interstitial tissue, marked by significant infiltration of the inflammatory cells: Lymphocytes, polymorphonuclear leukocytes (PMNs), and a limited number of eosinophils are observed in areas of infiltration between the tubular epithelial lining, resulting in tubulitis (H & E; X 400).

**Fig.3:**
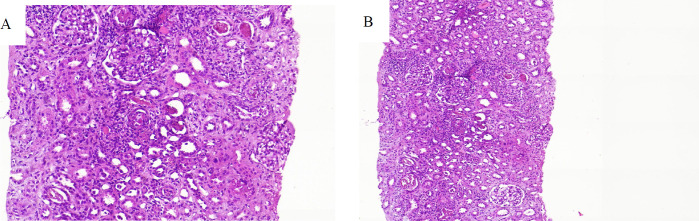
**A:**Histologic section showing severe acute tubulointerstitial nephritis, tubular injury, and cast nephropathy (red cell casts) with limited and focal acute thrombotic microangiopathy. (H & E; X 200,

 Many tubules contained red blood cells and red cell casts in which the cells were peripherally aggregated and the surrounding epithelium was destroyed. (Figure 3A) Focal interstitial deposition of hemosiderin was seen, and due to the intense inflammation, assessment of tubular atrophy or interstitial fibrosis was limited. A biopsy revealed one arteriole with intimal edema, neointimal proliferation, and a small thrombus. The large and interlobular arteries appeared unremarkable (Figures 1-3). On frozen tissue, immunofluorescence showed a negative immune reaction. Improvement in the laboratory tests gradually followed, and the patient's general condition was considered satisfactory. The patient was discharged on day 30. On discharge, a three-month course of tapering prednisolone treatment and weekly medical follow-up were ordered. After one month of follow-up, serum creatinine (Scr) and blood urea nitrogen (BUN) levels measured 0.55 and 16.4 mg/dl, respectively, while LDH had decreased to 458 IU/L.

**B:** There is intense inflammatory cell infiltration composed of lymphocytes, PMN, and few eosinophils with tubulitis that are diagnostic of acute tubulointerstitial nephritis (H&E; X 100)

## Discussion

The scorpion of old in Iran: From a historical perspective, scorpionism in Iran has a long history. The *H. lepturus* and *H. acanthocercus* belonging to the Hemiscorpiidae family and *Androctonus crassicauda* are the most dangerous scorpion species in the country (10, 11).

The first case of systemic injuries caused by *H. lepturus* (known locally as Gadeem) envenomation recorded in any literature is reported by the 10th and 11th-century Iranian physician Avicenna (9). *H. lepturus* accounts for 95 % of all scorpion sting deaths in Iran. *H. lepturus* venom is a complex mixture of peptides and enzymes, including heminecrolysin (which causes lysis of red blood cells), analgesic peptides, antimicrobial peptides, phospholipases (phospholipase D1 has been identified as a major neurotoxin), Hemicalcin (active on ryanodine-sensitive Ca2+ channels) and other cytotoxic agents (9, 12, 13).

Since 1998, this has been the subject of intense debate - the pathological mechanisms behind Hemolytic Uremic Syndrome (HUS) following a scorpion sting, particularly by *H. lepturus*. Several different hypotheses have been put forward that the venom from *H. lepturus* has a cytotoxic effect on vascular endothelial cells, causing them to become activated, aggregate, and eventually form a thrombus (1, 4). In HUS patients, a kidney pathology produced by this is known as thrombotic microangiopathy (TMA), which is marked by thickening of the arteriolar and capillary walls, edema of the capillary endothelium, and lifting up of the basement membrane (14, 15). The severe cases may present reduced glomerular filtration caused by renal cortical necrosis (1, 6).

Because the sting of *H. lepturus* is painless, the symptoms of the envenomation appear between 24- and 72 hours post-sting (3). According to one study, the delay in getting help before entering the hospital appears to be due to a lack of knowledge among the population about *H. lepturus* stings, with children being the ones who are mostly targeted and atypical symptoms occurring (1, 9). The most important part of managing *H. lepturus* stings is support, which depends on the patient and the specific injured system (6, 16). Most cases to date have led to Hemolytic Uremic Syndrome (HUS) (6, 16). A clinically confirmed diagnosis of HUS requires immediate initiation of treatment. Many studies have shown that hemodialysis, with or without the exchange of plasma using fresh frozen plasma (FFP), is an effective treatment for especially serious cases of atypical HUS caused by *H. lepturus* stings. Still, it is emphasized that further clinical trials must be done to verify this treatment approach. Moreover, symptomatic treatment is advised, including alkaline diuresis after hemoglobinuria and myoglobinuria, to prevent damage to the kidneys (1, 16).

According to a number of studies and case reports from the tropics, after being stung by *H. lepturus*, one may develop Disseminated Intravascular Coagulation (DIC), Acute Kidney Injury (AKI), and HUS. They may also require treatment. Valavi *et al.* have described pediatric cases in which HUS developed from an *H. lepturus* bite. The patient showed improvement after appropriate treatment, including daily plasma exchange and other symptomatic therapies. Severe cases of *H. lepturus* stings with HUS can be treated by hemodialysis. This has been demonstrated (1, 4). Renal histopathological examinations of postmortem specimens or biopsies of difficult cases show the same results as the current study (14, 15). A seven-year-old boy who developed HUS after being bitten by a scorpion had severe acute tubulointerstitial nephritis with tubular injury and cast nephropathy on kidney histopathology. In addition, areas of acute thrombotic microangiopathy were seen (5). 

## Conclusion

This case study clarifies that the venom of *H. lepturus* has systemic capability to cause serious injuries, including a HUS, in children and young individuals. Here, we emphasize the need for a thorough model to construct medical management guidelines for these cases in the tropical countries of Asia, Africa, and Europe.
